# SignalDetDDI: An SAS macro for detecting adverse drug-drug interactions in spontaneous reporting systems

**DOI:** 10.1371/journal.pone.0207487

**Published:** 2018-11-19

**Authors:** Masahiko Gosho, Tomohiro Ohigashi, Kazushi Maruo

**Affiliations:** 1 Department of Biostatistics, Faculty of Medicine, University of Tsukuba, Tsukuba, Japan; 2 Graduate School of Engineering, Tokyo University of Science, Tokyo, Japan; National Chiao Tung University College of Biological Science and Technology, TAIWAN

## Abstract

Statistical methods for detecting adverse drug reactions (ADRs) resulting from drug-drug interactions (DDIs) have been used in recent years to analyze the datasets in spontaneous reporting systems. We provide the SignalDetDDI macro in SAS to calculate the criteria for detecting ADRs resulting from the concomitant use of two drugs. We outline two criteria for detecting DDIs with the combination of two drugs and illustrate the implementation of the macro by way of an example. To implement the macro, a user specifies the target ADR and the two drugs to be evaluated. The SignalDetDDI macro outputs a table showing the number of reports on ADRs, the values of the two criteria for detecting ADRs, and the presence of DDIs. This macro enables users to easily and automatically assess the clinical DDIs that result from ADRs. The SignalDetDDI macro is freely available in the Supporting Information.

## Introduction

Polypharmacy, which is the use of multiple concomitant drugs to treat a medical condition, is recognized as a serious health, economical, and social problem. In particular, approximately 30% of elderly patients take six or more drugs concurrently [1]. The concomitant use of two or more drugs increases the risk of adverse drug reactions (ADRs). This problem is generally known as drug-drug interactions (DDIs), which are accurately defined as a “change in the effect of one drug in the presence of another drug, herbal medicine, food, drink, or environmental chemical agent” [2].

Spontaneous reporting systems are a crucial source of drug safety surveillance data and are commonly used to detect suspected ADRs and to generate the hypothesis of new ADRs in real-world settings. Authorities have been preparing databases for spontaneous reporting systems since the 1960s. Examples of these databases include: 1) the VigiBase, which is the world’s largest database, and is maintained by the WHO Collaborating Centre for International Drug Monitoring in Uppsala, Sweden; 2) the US Food and Drug Administration (FDA) Adverse Event Reporting System (FAERS), which is the database for products marketed in the US; 3) the EudraVigilance, which is a central database created by the European Medicines Agency in 2001; and 4) the Japanese Adverse Drug Event Report database, which is maintained by the Japanese Pharmaceuticals and Medical Devices Agency.

Statistical methods are needed to analyze ADRs resulting from the concomitant use of drugs, and to screen spontaneous reporting systems for possible DDI signals. Some researchers use a logistic regression model including the use of one drug *D*_1_ and another drug *D*_2_ as factors and an interactive term to indicate the concomitant use of both drugs [3, 4]. Thakrar et al. (2007) [5] also applied a multiplicative model (also known as a log-linear model) and an additive model (also referred to as a linear probability model) to general signals of DDIs by including an interactive term in the same manner as above. Computerized algorithms using these statistical methods were developed to detect DDIs automatically [6–8]. In addition, Norén et al. [9] developed a novel statistical method for DDI detection using the observed to expected ratio of the number of reports for ADRs for a combination of two drugs. This method is based on the 2.5th percentile of a disproportionality measure (*Ω*_025_), which is a shrinkage approach towards no association with the aim of reducing the false positive rate. Currently, the criterion *Ω*_025_ is frequently applied to databases to detect DDIs and computerized algorithms using this criterion are being developed for the automated detection of potential DDIs in spontaneous reporting systems [7, 8] Recently, Gosho et al. [10] proposed another statistical criterion to detect the DDIs that cause ADRs. The criterion is based on the chi-square statistics with Yates’s correction. Although Norén et al. [9] and Gosho et al. [10] introduced statistical methods for DDI detection and their properties, they did not mention the practical procedure and implementation of these methods to apply the databases for spontaneous reporting systems. Further, they did not show a concrete way to preprocess and clean datasets derived from the spontaneous reporting systems necessary for applying the methods. In addition, they did not provide computational algorithms to implement the methods using standard statistical software.

In this paper, we present a new SAS macro to calculate the values of the criteria proposed by Norén et al. [9] and Gosho et al. [10] for screening the ADRs resulting from the combined use of two drugs recorded in spontaneous reporting systems. We additionally provide SAS programs to preprocess and clean the datasets derived from the spontaneous reporting systems, which directly and automatically implement the methods for DDI detection. We also apply the macro to analyze the FAERS database. The use of this SAS macro enables the facile and automatic screening of clinical DDIs resulting in ADRs. The macro (SignalDetDDI) runs in the Microsoft Windows environment and requires SAS 9.3 (at least the SAS/BASE and the SAS/STAT components) or above.

## Design and implementation

### Definition of drug-drug interactions

A DDI is defined as occurring in a situation in which “the effects of one drug, *D*_1_, increase in the presence of another drug, *D*_2_.” The interaction is denoted as the interdependence between the effects of two drugs, and is considered to be synergistic [9]. The interaction is exclusive of the additive effects of the two drugs. Our focus in this study was to detect whether synergistic interactions occurred between two drugs.

Let “ADR A” denote an adverse drug reaction “A” of interest. *n*_111_ represents the number of reports on ADR A listing both *D*_1_ and *D*_2_, *n*_101_ denotes the number of reports on ADR A listing *D*_1_, but not *D*_2_, *n*_011_ signifies the number of reports on ADR A listing *D*_2_, but not *D*_1_, and *n*_001_ denotes the number of reports on ADR A in the absence of both *D*_1_ and *D*_2_. The incidence of probability of ADR A, the number of ADR reports excluding ADR A, and the total number of reports are listed in Table 1.

**Table 1:** Four-by-two contingency table for DDI evaluation.

Number of events (Incidence probability for ADR A)	ADR A	Not ADR A	Total
Neither *D*_1_ nor *D*_2_	*n*_001_ (*p*_00_)	*n*_000_	*n*_00⋅_
Only *D*_1_	*n*_101_ (*p*_10_)	*n*_100_	*n*_10⋅_
Only *D*_2_	*n*_011_ (*p*_01_)	*n*_010_	*n*_01⋅_
*D*_1_ and *D*_2_	*n*_111_ (*p*_11_)	*n*_110_	*n*_11⋅_

We focused on the additive assumption for DDIs [5]. When no interaction is detected under the additive assumption, the excess risk associated with *D*_1_ in the absence of *D*_2_ is the same as the excess risk associated with *D*_1_ in the presence of *D*_2_. In this case, $RD_{D_{1}D_{2}}=RD_{D_{1}}+RD_{D_{2}}$, where $RD_{D_{1}}=p_{10}-p_{00}$, $RD_{D_{2}}=p_{01}-p_{00}$, and $RD_{D_{1}D_{2}}=p_{11}-p_{00}$. Thus, *p*_11_ − *p*_10_ − *p*_01_+ *p*_00_ = 0. In the event of a positive interaction, the assumption is that this measure is greater than 0. The criteria proposed by Norén et al. [9] and Gosho et al. [10] are structured with the purpose of detecting the additive risk.

### Criteria for detecting adverse drug reactions

Norén et al. [9] proposed a criterion to detect suspected DDIs in databases containing spontaneous reports. This method is based on a measure calculated as the ratio of the observed reporting rate, *f*_11_, of an ADR resulting from the combination of two drugs and its expected value, *E*[*f*_11_]. The expected value is always unknown and should be estimated.

Let

$$\begin{array}{c} f_{00}=\frac{n_{001}}{n_{00\cdot }},\;f_{10}=\frac{n_{101}}{n_{10\cdot }},\;f_{01}=\frac{n_{011}}{n_{01\cdot }},\;f_{11}=\frac{n_{111}}{n_{11\cdot }} \end{array}$$

The estimator *g*_11_ of *E*[*f*_11_] is given as follows [9]:

$$\begin{array}{c} g_{11}=1-\frac{1}{\text{max}(\frac{f_{00}}{1-f_{00}},\frac{f_{10}}{1-f_{10}})+\text{max}(\frac{f_{00}}{1-f_{00}},\frac{f_{01}}{1-f_{01}})-\frac{f_{00}}{1-f_{00}}+1} \end{array}$$ (1)

Norén et al. [9] provided a measure for detecting ADRs with two drugs:

$$\begin{array}{c} \Omega ={\text{log}}_{2}\frac{n_{11}+0.5}{E_{111}+0.5}, \end{array}$$ (2)

where *E*_111_ = *g*_11_*n*_11⋅_. The Ω measure can be motivated from both frequentist and Bayesian perspectives. In the frequentist approach, the measure for signal detection is given as follows:

$$\begin{array}{c} \Omega_{025}=\Omega -\frac{\phi (0.975)}{\ln\left(2\right)\sqrt{n_{111}}}, \end{array}$$ (3)

where *ϕ*(0.975) is the 97.5th percentile of the standard normal distribution. With the Bayesian approach, the lower limit, Ω_025_, of the exact two-sided 95% credibility interval for Ω in Eq (2) can be numerically estimated [9]. In both the frequentist and Bayesian approaches, Ω_025_ > 0 is used as a threshold to screen for signal under the combination of two drugs [9], with Ω_025_ for these two approaches denoted as Ω_025*F*_ and Ω_025*B*_, respectively.

Gosho et al. [10] proposed another criterion to screen the ADR resulting from the concomitant use of two drugs. Gosho et al. [10] prepared the following measure, *χ*, to estimate the discrepancy between the observed and expected number of events with specific drug combinations:

$$\begin{array}{c} \chi =\frac{n_{111}-E_{111}-0.5}{\sqrt{E_{111}}} \end{array}$$ (4)

The expected number of events, *E*_111_, can be estimated using Eq (1) and *χ* is the square root of the chi-square test statistics with a corretion term “0.5”. In Eq (4), *χ* is the criterion that directly measures the discrepancy between the observed and expected number of reports in drug combinations to which Yates’s correction has been applied to avoid the small-sample issue. Ultimately, *χ* > 2 is used as a threshold to screen for a signal resulting from the concomitant use of two drugs.

The methods proposed by Norén et al. [9] and Gosho et al. [10] focus on the detection of synergistic interaction effects. Inversely, these methods are not designed to detect antagonistic effects. Thus, our SAS macro (SignalDetDDI) highlights only the synergistic rather than the antagonistic effects.

### Working with the macro

The SignalDetDDI macro is included with this article in the file SignalDetDDI.sas. The arguments taken by the macro are summarized in Table 2. As shown in the table, the names of datasets for ADRs and drugs that are generated from the database in spontaneous reporting systems (AE_INDS and DRUG_INDS, respectively), the name of the dataset that stores an ADR and the two drugs to be analyzed for signal detection (LIST_INDS), the name of a variable identifying each patient (ID), the names of variables for the ADR and drug (AE_NAME and DRUG_NAME, respectively), and the name of a variable representing the sequence in each patient (DRUG_SEQ), should be specified for implementing the macro.

**Table 2:** Arguments for implementing SignalDetDDI macro.

Argument	Description	Note
AE_INDS	Name of the SAS dataset storing ADRs	The dataset can be SAS. The data structure is illustrated in Table 3.
DRUG_INDS	Name of the SAS dataset storing drugs	The dataset can be SAS. The data structure is illustrated in Table 4.
LIST_INDS	Name of the SAS dataset storing the ADR and two drugs	An ADR and two drugs with one or more record to be analyzed for signal detection, can be specified. The dataset can be SAS. The data structure is illustrated in Table 5.
ID	Name of the variable identifying each patient	Character and numerical types are available.
AE_NAME	Name of the ADR variable	
DRUG_NAME	Name of the drug variable	
DRUG_SEQ	Name of the sequence variable (within a patient)	Character and numerical types are available.

The SignalDetDDI macro consists of three nested macros, DataHandling, SignalDS, and Signal. The DataHandling macro generates the analysis datasets using the datasets specified at AE_INDS and DRUG_INDS, for signal detection. The SignalDS macro calculates the number of reports shown in Table 1, i.e., *n*_001_, *n*_000_, *n*_101_, *n*_100_, *n*_011_, *n*_010_, *n*_111_, and *n*_110_, in the case when an ADR and the use of two drugs are identified. The Signal macro provides the values of the criteria (Ω_025*F*_, Ω_025*B*_, and *χ*) for screening the ADRs resulting from the concomitant use of two drugs.

### Datasets for implementation of the macro

A user can call the SignalDetDDI macro by inputting the arguments listed in Table 2. Before the user implements the macro, they would need to prepare three SAS datasets in which to store the ADRs, drug, and the target ADR and the two drugs to be evaluated, respectively.

The ADRs are stored in aeds, which is a dataset provided by the FAERS database and is partially reproduced in Table 3. The aeds data include the case number, primaryid; a within-subject observation identifier, ae_seq; and the name of the ADRs reported, ae. The identifier ae_seq is not necessary for implementing the macro.

**Table 3:** Example of a dataset of three patients for the aeds data.

primaryid	ae_seq	ae
100033062	1	Cough
100033062	2	Throat irritation
100033073	1	Rhinorrhoea
100033083	1	Malaise
100033083	2	Unevaluable event

The drugds, which is a dataset provided by the FAERS database, is used to store the drug, and is partially reproduced in Table 4. The drugds data include the case number, primaryid; a within-subject observation identifier, drug_seq; and the name of drugs reported, drug. All three variables are necessary for implementing the macro.

**Table 4:** Example of a dataset of three patients for the drugds data.

primaryid	drug_seq	drug
100033062	1	LETAIRIS
100033062	2	TYVASO
100033073	1	LETAIRIS
100033073	2	LETAIRIS
100033083	1	LETAIRIS
100033083	2	LETAIRIS

The listds data in Table 5 store the target ADR adr and the two drugs d1 and d2 to be evaluated. All three variables are necessary for implementing the macro. This dataset can contain data originating from one or more records.

**Table 5:** Example of a dataset for analyzing the ADR (adr) for the concomitant use of two drugs (d1 and d2) for the listds data.

adr	d1	d2
hypoglycaemia	sitagliptin	nateglinide
hypoglycaemia	sitagliptin	repaglinide
hypoglycaemia	sitagliptin	miglitol

### Output of the macro

The macro creates an output table that includes *n*_001_, *n*_000_, *n*_101_, *n*_100_, *n*_011_, *n*_010_, *n*_111_, and *n*_110_ in Table 1, where the ADR and two drugs are specified by the user. The output table also contains the value of the criteria (Ω_025*F*_, Ω_025*B*_, and *χ*) for screening the ADR for the concomitant use of the two drugs and the decision flag of the signal. The flag has the value “Y” or “N” to indicate whether the signal is detected using the three criteria. The details of the output are shown in the Results section.

## Results

We applied the SignalDetDDI macro to the FAERS and evaluated the risk of hypoglycaemia as an ADR (adr) for the three combinations of two diabetic drugs (d1 as d2) shown in Table 5. Drug-induced hypoglycemia is the commonest adverse event and the most important complication of antidiabetic medications. DDIs are also one of the exogenous risk factors of iatrogenic hypoglycemia (e.g., [11, 12]). Sitagliptin is a selective dipeptidyl peptidase-4 inhibitor for the treatment of patients with type 2 diabetes and is sometimes used in combination with other classes of antidiabetic agents such as glinides (e.g., mitiglinide and repaglinide) and *α*-glycosidase inhibitors (e.g., miglitol).

### Datasets

The FAERS is a database that contains reports about adverse events and medication errors, as well as complaints about product quality resulting in adverse events that were submitted to the FDA [13]. The database is designed to support the FDA’s post-marketing safety surveillance program for drug and therapeutic biologic products. Adverse events and medication errors are coded using the terms in the Medical Dictionary for Regulatory Activities (MedDRA) terminology [13]. The database is managed by the FDA, contains information that has been entered since 2004, and is updated quarterly. The structure of the database basically complies with the international safety reporting guidelines (International Conference on Harmonization E2B). It comprises seven data files: patient demographics, drug information, indication, case outcome, reactions/events, report source, and therapy. The name of the ADR is defined in the MedDRA as the preferred term. Standardized MedDRA Queries (SMQs) are not supported in the database. The drug name is a combination of the brand (trade), generic, and general names of drugs.

We provide the supportive SAS program file Supplement macro for handling FAERS data files.sas in S1 Appendix. The file includes four SAS macros. The first macro is designed to prepare the SAS datasets by the indicated time ranges and converts the FAERS raw data files posted on the FDA website into the SAS datasets. The raw data files can be downloaded from the website [13]. The user can implement the macro by specifying the period of interest for which they require data from the database. The second macro allows the aeds and drugds datasets to be prepared for implementation of the SignalDetDDI macro. The macro also combines the datasets according to each period derived from the first macro. The third and fourth are example macros that replace the brand name(s) of a target drug with the general name in the drugds dataset for the implementation of the SignalDetDDI macro. Before implementing these macros, the user needs to specify the general (ingredient) name of the two target drugs to be analyzed. Using the RxNav system based on RxNorm (https://mor.nlm.nih.gov/RxNav/), the names of each drug are tabulated as a CSV file. After storing the CSV files in an appropriate folder, the user can implement the third macro by specifying the names of each CSV file and the pass name in which the files are stored. After implementing the third macro, the user can replace the brand name(s) with the general name by using the forth macro.

We downloaded data from the FAERS database for the period 2017 Q1 to Q4 via the FDA website [13] and generated the aeds and drugds datasets using the Supplement macro for handling FAERS data files, which we then analyzed. The aeds dataset contains 4,017,430 adverse reactions and drugds contains 4,863,924 drug information records.

### Implementation of the macro

The implementation of the SignalDetDDI macro is demonstrated using the aeds, drugds, and lisitds data. The code for implementing the SignalDetDDI macro, code for calling the macro and generating the listds, and code for generating the aeds and drugds (Supplement macro for handling FAERS data files) are given in S1 Appendix. The user implements the SignalDetDDI macro by inputting values for the various arguments as shown in the code below (also see Table 2) and invokes the macro.

%SignalDetDDI (AE_INDS = aeds, DRUG_INDS = drugds, LIST_INDS = listds, ID = primaryid, AE_NAME = ae, DRUG_NAME = drug, DRUG_SEQ = drug_seq);

### Output and result

Table 6 shows the output results of the macro. In Table 6, “ADR” is the specified ADRs, and “Drug1” and “Drug2” are the names of the two drugs to be analyzed for detecting the ADRs. Further, “n000”, “n001”, “n100”, “n101”, “n010”, “n011”, “n110”, and “n111” are the number of reports for the ADRs and two drugs defined in Table 1, “E111” is the estimated *E*_111_ in Eq (1), and “Omega025F,” “Omega025B,” and “Chi” refer to the criteria values for signal detection given in Section 1. When the criteria values cannot be calculated, they are displayed as “NC.” “Signal by Omega025F?,” “Signal by Omega025B?,” and “Signal by Chi?” are the decision of the signal using the three criteria, Ω_025*F*_, Ω_025*B*_, and *χ*, respectively. If the signal is detected, then the value is “Y”, otherwise it is “N.” As mentioned earlier, when Ω_025*F*_ and Ω_025*B*_ are greater than 0 and *χ* is greater than 2, a signal is considered to have been detected. Finally, “Warning” is a message that is output only when the criteria values could not be calculated and displays the reason why the values were not calculated.

**Table 6:** Outputs of analysis for implementing the SignalDetDDI macro.

ADR	Drug1	Drug2	n000	n001	n100	n101	n010	n011	n110	n111	E111
hypoglycaemia	sitagliptin	nateglinide	1360164	3005	6677	105	168	0	21	5	0.40
hypoglycaemia	sitagliptin	repaglinide	1359906	2941	6639	109	426	64	59	1	8.48
hypoglycaemia	sitagliptin	miglitol	1360292	3003	6696	110	40	2	2	0	0.12

Omega025F	Omega025B	Chi	Signal by Omega025F?	Signal by Omega025B?	Signal by Chi?	Warning
1.34	1.25	6.46	Y	Y	Y	
−5.41	−6.23	−2.74	N	N	N	
NC	NC	NC	N	N	N	*n*_111_ = 0

In this example, the concomitant use of sitagliptin and nateglinide was noted using the Ω_025*F*_, Ω_025*B*_, and *χ* for hypoglycaemia. The criteria values for Ω_025*F*_, Ω_025*B*_, and *χ* resulting from the concomitant use of sitagliptin and miglitol cannot be calculated because “n111” (*n*_111_)’, the number of reports of hypoglycaemia using both of these two drugs, is zero. As the subset of the database from the FAERS used in this analysis is quite small, the results should be considered illustrative; thus, one should not infer that this result is obviously invalid.

## Availability and future directions

The research presented in this paper led to the development of the SignalDetDDI macro to calculate the values of the criteria (Ω_025*F*_, Ω_025*B*_, and *χ*) for detecting clinical adverse DDIs in spontaneous reporting systems. All the SAS codes are freely available (S1 Appendix). This macro enables researchers of pharmacoepidemiology and drug safety to analyze big data from spontaneous reporting systems in a facile and automatic manner. In any signal detection analysis, the risk of false positives and false negatives for adverse DDIs exists. In other words, a signal for DDIs is either falsely detected or missed. It should be kept in mind that the false positive and false negative rates cannot be zero simultaneously.

The SignalDetDDI macro has the following practical limitations. Before the macro can be implemented, the user would have to preprocess and clean the datasets aeds and drugds because databases in spontaneous reporting systems provided by the authorities are typically not subjected to data management. For example, these databases often include a mixture of brand (trade), generic, and general names of drugs. TheSupplement macro for handling FAERS data files.sas is helpful to convert brand names to general names. Another limitation is that drugs that aim to inhibit adverse effects of other drugs may be difficult to distinguish in this system (e.g., anticancer drugs and anti-emetics). Moreover, this macro cannot be directly used to screen DDIs among three or more drugs.

## Supporting information

S1 Appendix All SAS codes are provided. (ZIP)
